# Proprioceptive assessment in clinical settings: Evaluation of joint position sense in upper limb post-stroke using a robotic manipulator

**DOI:** 10.1371/journal.pone.0183257

**Published:** 2017-11-21

**Authors:** Sara Contu, Asif Hussain, Simone Kager, Aamani Budhota, Vishwanath A. Deshmukh, Christopher W. K. Kuah, Lester H. L. Yam, Liming Xiang, Karen S. G. Chua, Lorenzo Masia, Domenico Campolo

**Affiliations:** 1 Robotics Research Centre, School of Mechanical and Aerospace Engineering, Nanyang Technological University, Singapore, Singapore; 2 NUS Graduate School for Integrative Sciences and Engineering, National University of Singapore, Singapore, Singapore; 3 Interdisciplinary Graduate School, Nanyang Technological University, Singapore, Singapore; 4 Centre for Advanced Rehabilitation Therapeutics, Department of Rehabilitation Medicine, Tan Tock Seng Hospital, Singapore, Singapore; 5 School of Physical and Mathematical Sciences, Nanyang Technological University, Singapore, Singapore; University of Ottawa, CANADA

## Abstract

Proprioception is a critical component for motor functions and directly affects motor learning after neurological injuries. Conventional methods for its assessment are generally ordinal in nature and hence lack sensitivity. Robotic devices designed to promote sensorimotor learning can potentially provide quantitative precise, accurate, and reliable assessments of sensory impairments. In this paper, we investigate the clinical applicability and validity of using a planar 2 degrees of freedom robot to quantitatively assess proprioceptive deficits in post-stroke participants. Nine stroke survivors and nine healthy subjects participated in the study. Participants’ hand was passively moved to the target position guided by the H-Man robot (Criterion movement) and were asked to indicate during a second passive movement towards the same target (Matching movement) when they felt that they matched the target position. The assessment was carried out on a planar surface for movements in the forward and oblique directions in the contralateral and ipsilateral sides of the tested arm. The matching performance was evaluated in terms of error magnitude (absolute and signed) and its variability. Stroke patients showed higher variability in the estimation of the target position compared to the healthy participants. Further, an effect of target was found, with lower absolute errors in the contralateral side. Pairwise comparison between individual stroke participant and control participants showed significant proprioceptive deficits in two patients. The proposed assessment of passive joint position sense was inherently simple and all participants, regardless of motor impairment level, could complete it in less than 10 minutes. Therefore, the method can potentially be carried out to detect changes in proprioceptive deficits in clinical settings.

## Introduction

The high demand for neurorehabilitation has stimulated interest for technology-assisted systems with the intention to decrease the therapist’s workload and to facilitate recovery at an affordable cost [1–3]. As a result, several robotic solutions have been developed in the last couple of decades to promote sensory motor learning [4–7]. To date, clinical studies with these setups have shown that robot-assisted therapy of the upper-extremity is at least as effective as conventional rehabilitation therapy in reducing motor impairments [3, 8, 9]. However, an arguably equal, if not more important role of technology as an assessment tool has been explored less thoroughly. In particular, a limited amount of work has been done in evaluating somatosensory integrity, which plays a critical role in performing activities of daily living as known for the last few decades. Rothwell in the early 80’s studied manual motor function in a deafferented man with severe sensory loss and found that the subject could perform trained complex tasks even with sensory loss, but could not translate this skill to other similar activities due to limited learning—a potential consequence of lack of sensory feedback [10]. Hence, a clear understanding of somatosensory loss is critical for identification of individual’s specific therapy regimes.

Sensory dysfunction can affect tactile sensation, temperature discrimination and proprioception [11, 12]. Proprioception, the sense of body position in space, encompasses the perception of joint position, active and passive movement (kinesthesia), force and effort [13]. It is a critical component for motor control and learning and allows the generation of smooth and coordinated movements [14]. Stroke patients presenting defective proprioceptive abilities may have difficulties in estimating the position of their limbs and maintaining them in a steady posture in absence of vision [10], resulting in negative implications for safety [15] leading to a deterioration of their quality of life. This potentially also results in difficulties in learning novel movements, and difficulties in improving the quality of movements over time with functional repetitions [16].

Conventionally used clinical scales for assessment of proprioception are generally ordinal in nature and do not provide a clear distinction between different subcomponents of proprioception. As a consequence, contrasting results have been obtained in the quantification of proprioceptive deficits in stroke patients. For example, proprioceptive assessment performed by mean of the Rivermead assessment of somatosensory performance (RASP) resulted in the identification of deficits in the 27% of 93 acute stroke patients screened, with no difference between arm and leg proprioception [12]; while another study involving 70 patients screened using the Nottingham sensory assessment (NSA) reported impairments that ranged from the 34% at the ankle to 63% at the shoulder [17].

In order to develop a better understanding of proprioceptive defects and their impact on motor functions, different quantitative assessment routines have been proposed in recent years using technological solutions [18–22]. These paradigms quantify proprioceptive deficits based on movement detection [23–25], discrimination [26] and position matching [21, 27]. In this paper, we focus on the latter, i.e. position matching, which has been shown to be an effective approach for assessment of change in proprioceptive acuity [16]. However, due to practical implementation issues including time constraints, equipment required and cost, it is yet to be established in clinical settings with stroke participants.

As a step in this direction, the goal of this paper was to investigate the clinical applicability and validity of a refined joint matching task to quantitatively assess proprioceptive deficits in post-stroke participants, keeping in consideration the practical limitations of clinical settings. We selected the passive joint matching approach, designed to analyze the ability to match the passive distance traveled by the limb to account for limited motor functions of stroke patients [28]. The time duration of the complete assessment was set to around 10 minutes, such that it can be integrated as a part of other motor and sensory assessments. Finally, the whole study was carried out using a portable and low-cost 2 degrees of freedom (DOF) manipulator, which can also be used for motor assessment and training of sensorimotor functions, hence limiting the need for additional equipment.

## Materials and methods

### Participants

Nine stroke survivors (5 males, mean age±SD: 53.7±7.3 years), recruited for a randomized control trial (ClinicalTrials.gov ID: NCT02188628), consented to undergo the proprioceptive test during the week 0 (baseline) motor assessment. The goal of the randomized control trial was to compare differences in clinical outcomes at pre and post treatment of patients undergoing 1.5 hours of conventional therapy versus one hour of robotic training followed by 30 minutes of conventional therapy. Recruitment was conducted over a period of about 10 weeks (starting from 1^st^ of July 2016 to 9^th^ September 2016). Patients who were screened for eligibility were those under the care of the Rehabilitation Department of Tan Tock Seng Hospital, Singapore. A total of 12 patients were invited for screening and nine met the following inclusion criteria for participating in the study: (1) diagnosis of a single stroke (ischaemic or haemorrhagic) confirmed by brain imaging; (2) post-stroke duration above 3 months; (3) age between 21 and 85 years; (4) hemiplegic pattern of arm motor impairment with shoulder abduction motor power ≥ 3/5 and elbow flexion motor power ≥ 3/5 measured with the Medical Research Council (MRC) Scale for Muscle Strength; (5) Fugl-Meyer, arm section (FMA) motor subscore above 20 and (6) presence of motor incoordination or motor ataxia. Patients were excluded if they exhibited cognitive impairments or uncontrolled behaviour (Folstein mini mental state exam MMSE <26/30). As reported in Table 1, two patients had a history of right-hemisphere stroke while the others had left-hemisphere damage and the FMA score of all participants ranged between 22 and 58 (mean value±SD: 43.4±13.1). Prior to recruitment, patients signed the informed consent form which conformed to the ethical standards expressed in the 1964 Declaration of Helsinki. The individual in this manuscript has given written informed consent (as outlined in PLOS consent form) to publish these case details. The study methodology was approved by the Domain Specific Review Board of the National Healthcare Group (NHG).

**Table 1:** Stroke patients characteristics.

Subject ID	Age (years)	Gender	Handedness	Time since onset (months)	Nature (Haemorrhagic or Ischaemic)	Paretic Arm	FMA (0-66)
S1	52	F	R	23	H	R	43
S2	67	M	R	23	I	R	58
S3	56	M	R	5	I	L	40
S4	53	F	L	21	H	L	53
S5	57	F	R	25	H	R	39
S6	42	M	R	15	H	R	56
S7	45	M	R	8	H	R	26
S8	53	F	R	8	H	R	54
S9	58	M	R	5	H	R	22

Nine right-handed subjects (3 males, mean age±SD: 54.8±3.9 years old) with no previous history of neurological, psychiatric or neuromuscular disorders formed the control group. All participants were informed about the experiment and provided written informed consent. The experiment was approved by the Institutional Review Board at Nanyang Technological University.

### Experimental setup

H-Man, a planar robotic manipulandum designed for the rehabilitation and training of upper-limb sensorimotor functions was used in the experiments [29]. It is actuated by two motors in a differential configuration that drive two perpendicular linear sliders upon which a handle is attached. The motors can provide forces of up to 30 N at the end-effector in any specified direction in the workspace. These forces are used to assist or resist the motion of the user. H-Man has a homogeneous behavior across its workspace and present low friction and low inertia. It is easy to control, lightweight (about 7kg) and intrinsically safe.

The apparatus is integrated with a PC equipped with a real-time control unit, Quanser QPIDe, which acquires the signal from the encoders and commands the values of the motor torques through QUARC blocks in a Simulink model. The same model manages the communication via transmission control protocol (TCP/IP) between the real-time system and a visual interface designed in the Unity game engine. The visual outputs are displayed on a 20” computer monitor. The interaction with H-Man is simplified by a graphical user interface based on the MATLAB GUI-Guide tool which allows the selection of multiple assessment tasks or training schemes for rehabilitation.

### Procedure

**Fig 1:**
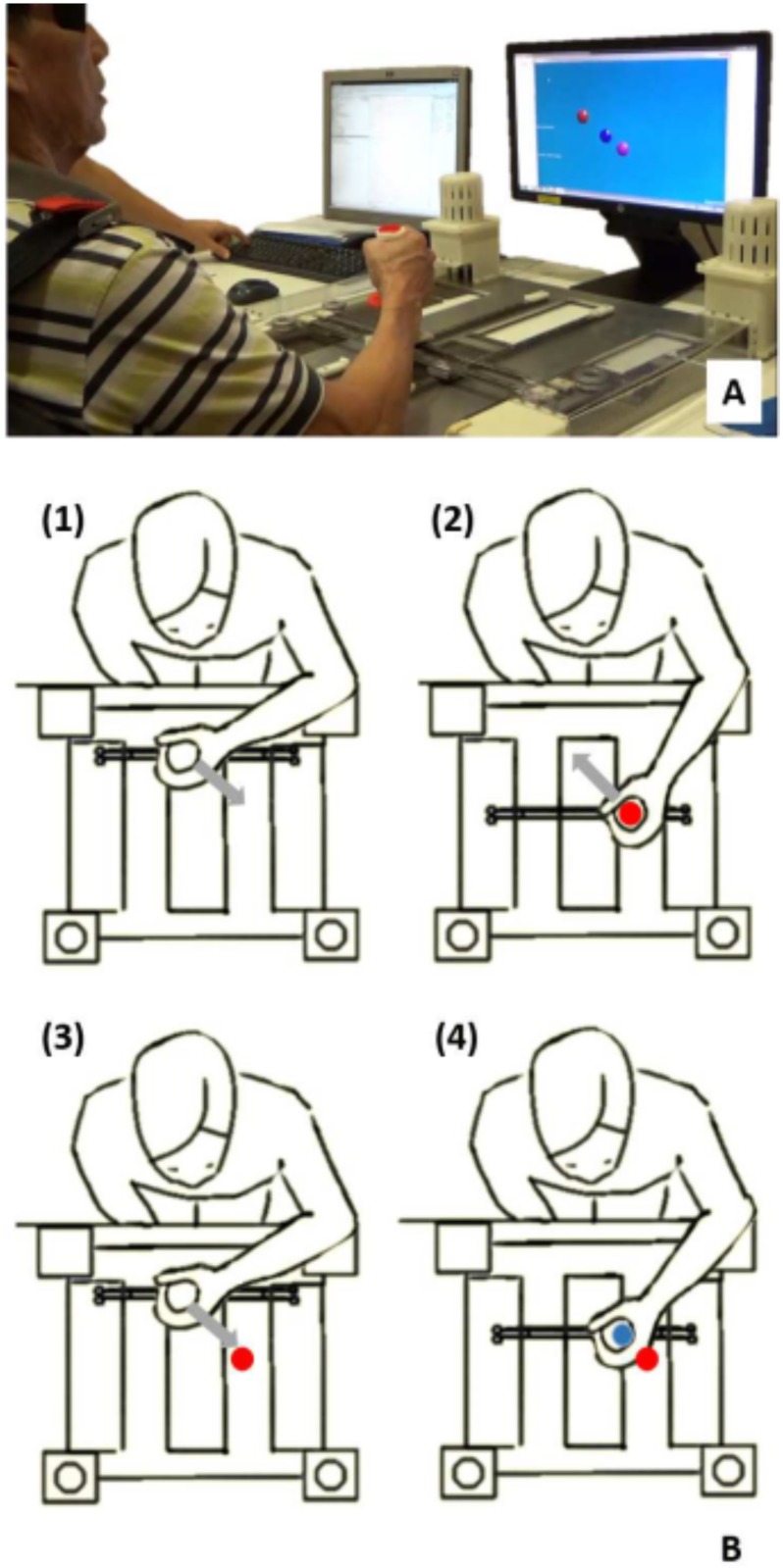
Apparatus and procedure. (A) Participant (blindfolded) holding the handle of H-Man, the robotic device employed in the study. (B) Experimental procedure: starting from the initial position (1), H-Man placed the handle on the target position and held it there for 2 seconds (2), after which the handle was returned to the initial position to start the new movement towards the same target (3), which was stopped via a hand-held button by the investigator when the participant verbally indicated that the position of the handle matched the target (4). The handle was then returned to the initial position for the following trial.

Participants were seated in a height-adjustable chair in front of the robotic manipulandum, so that the center of the sternum was aligned with the handle of the H-Man robot (Fig 1A). The distance between the initial position of the handle and the sternum was set to 25 cm. The participants’ elbow was initially bent at approximately 90° and their impaired wrist (or the dominant one in case of healthy participants) was placed on the handle in a comfortable position. To avoid compensatory movements typical in stroke patients, shoulder straps attached to the chair were used to maintain their trunk in a static position, while allowing rotation of the shoulder and elbow joints. The stroke patients’ hand was strapped to the handle if proper grasp was not possible. All participants were instructed to face forward, to keep their eyes closed (or blindfolded, if requested by the participant), and to keep the arm muscles relaxed during the whole duration of the experiment, which involved position estimation with a memory component.

In particular, in order to measure the ability to match the perceived position to a predefined target, the participant’s limb was moved by the robot at a constant velocity of 7 cm/s to the target position by the H-Man robot (*Criterion movement*), and held there for 2 seconds so that the subject could memorize the position. The manipulator then returned to the starting position at the same velocity. The manipulandum then moved the limb again towards the target at a constant velocity of 2 cm/s (*Matching movement*) until the subject felt that the original target position was reached. At this occurrence, the subject was asked to verbally notify the investigator, who would then halt the movement via a hand-held button. The handle was then returned by the robot to the initial position to begin a new trial (Fig 1B).

The assessment was carried out for movements in three directions: forward, oblique in the contralateral side and oblique in the ipsilateral side, with reference to the affected arm for the stroke group or the dominant arm for the control group. For each trial, the target location was pseudo-randomly chosen among three possible positions at a fixed distance of 10 cm from the starting position but in different directions: -45°, 0°, and +45° (targets are called respectively, contralateral, central, and ipsilateral when testing the right arm). The complete assessment consisted of 30 trials, 10 in each direction for the control group and 18 trials, six in each direction, for the stroke group. The smaller number of trials for this group resulted in a shorter assessment that prevented fatigue but that was still suitable to detect proprioceptive deficits [30]. No feedback about participant’s performance was given during the experiment to avoid recalibration.

### Analysis

#### Metrics

Three task performance metrics were adopted from the literature to assess participants level of proprioceptive acuity [16]. *Absolute error* was defined as the absolute matching difference between the final position of the end effector during the Matching movement *F*_*i*_ = (*x*_*i*_, *y*_*i*_) and the target position displayed in the Criterion movement *T*_*j*_ = (*x*_*Tj*_, *y*_*Tj*_):

$$\begin{array}{c} Absolute\;{Error}_{j,i}=\left|T_{j,i}-F_{j,i}\right| \end{array}$$ (1)

$$\begin{array}{c} Absolute\;{Error}_{j}=median\{Absolute\;{Error}_{j,1},\cdots ,Absolute\;{Error}_{j,N}\} \end{array}$$ (2)

Here, *i* represents the trial (*i* = 1,2..*N*, where *N* = 10 for the control group and *N* = 6 for the stroke group) and *j* represents the target for which the trial was carried out (*j* = 1 for contralateral, *j* = 2 for central and *j* = 3 for ipsilateral).

*Signed error* was defined as the difference between the Euclidean norm of the target position and the matching movement

$$\begin{array}{c} Signed\;{Error}_{j,i}=∥T_{j}∥-∥F_{j,i}∥ \end{array}$$ (3)

$$\begin{array}{c} Signed\;{Error}_{j}=median\{Signed\;{Error}_{j,1},\cdots ,Signed\;{Error}_{j,N}\} \end{array}$$ (4)

*Variability*, defined as the standard deviation of the signed error, was used as a measure of precision

$$\begin{array}{c} Variability_{j}=SD\{Signed\;{Error}_{j,1},\cdots ,Signed\;{Error}_{j,N}\} \end{array}$$ (5)

For absolute and signed error, the median was chosen to limit the influence of outlying values.

#### Statistical analysis

To test the clinical applicability of the proposed methodology, we analyzed differences between control and stroke group using a mixed ANOVA (between-subject factor Group: control and stroke, within-subject factor Target: contralateral, central, ipsilateral) on the group data for absolute and signed errors and variability. Statistical significance was considered for *p*-values lower than 0.05. Post-hoc analysis on significant effects was performed using Bonferroni-corrected paired t-tests. To evaluate the validity of the proposed methodology for the detection of proprioceptive impairments, pairwise comparisons, with the significance level adjusted (*p*-values lower than 0.0056), were run between each stroke participant’s absolute errors in the 6 trials and the 9 absolute errors of the control group in each direction. Pairwise comparisons were also run between each stroke participant’s signed errors in the 6 trials and the 9 signed errors of the control group in each direction.

## Results

The dataset containing the signed errors of healthy participants and stroke patients in each trial are reported in S1 and S2 Datasets respectively.

### Absolute errors

**Fig 2:**
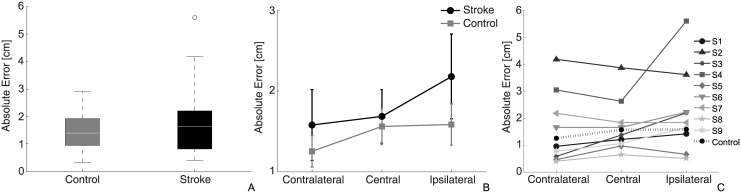
Absolute errors. (A) Box-plot of absolute errors for the two groups. (B) Mean absolute errors in the 3 directions for the two groups, where the gray squares represent data from the control group and black circles represent stroke patients. (C) Absolute errors for each patient and mean value of the control subjects.

Overall, the mean absolute error of the control group (mean value±SEM: 1.5±0.1 cm) was smaller and not significantly different from that of stroke patients (1.8±0.3 cm), *F*_(1,16)_ = 0.51, *p* = 0.49 (Fig 2A). Analysis revealed a significant effect of Target on the absolute error, Greenhouse-Geisser corrected *F*_(1.28,20.45)_ = 5.60, *p* = 0.02. No interaction effect between Group and Target was found, *F*_(1.28,20.55)_ = 0.88, *p* = 0.39: regardless of the group, the contralateral target was matched with the highest accuracy, with an error of 1.2±0.4 cm and 1.6±0.4 cm for control and stroke group respectively. The central target was matched with a mean error of 1.6±0.3 cm (control group) and 1.7±0.3 cm (stroke group), while the highest error was found for the ipsilateral target: 1.7±0.4 cm and 2.2±0.4 cm. In all cases, the mean absolute control error tended to be smaller than stroke participants (but was not significant). Post-hoc analysis revealed a significant difference between the central and contralateral (mean difference±SEM = 0.2±0.1 cm, *p* = 0.04), and between controlateral and ipsilateral (mean difference±SEM = 0.5±0.2 cm, *p* = 0.03) as shown in Fig 2B.

Analysis of differences between individual stroke participants and the control group showed that two participants, S2 and S4, were significantly different from the control group (Fig 2C). Mean differences between control and subjects are reported in Table 2, showing significant higher errors for the contralateral and ipsilateral targets for patient S4 and for the contralateral and central targets for patient S2.

**Table 2:** Pairwise comparisons of absolute errors between each subject and the control group in the 3 directions. Mean differences are evaluated as Control-S. Significant differences are marked with an asterisk.

	Contralateral		Central		Ipsilateral	
S	Mean Difference	p-value	Mean Difference	p-value	Mean Difference	p-value
1	0.1	0.76	0.6	0.34	0.3	0.64
2	-3.0	<0.005*	-2.6	<0.005*	-2.1	0.01
3	0.6	0.14	-0.2	0.73	-0.3	0.66
4	-1.4	<0.005*	-1.0	0.10	-2.9	<0.005*
5	0.8	0.03	0.4	0.54	0.4	0.56
6	-0.2	0.64	-0.1	0.88	-0.6	0.42
7	-0.7	0.07	-0.5	0.35	-0.1	0.90
8	0.9	0.02	0.8	0.16	1.1	0.15
9	0.3	0.36	0.3	0.56	-0.1	0.94

### Signed errors

**Fig 3:**
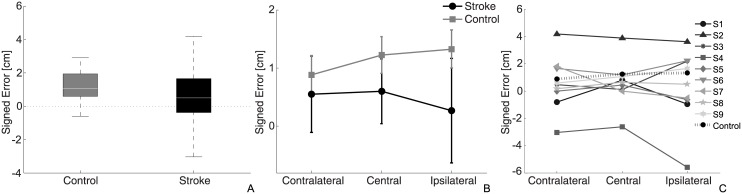
Signed errors. (A) Box-plot of signed errors for the two groups. (B) Mean signed errors in the 3 directions for the two groups, where the gray squares represent data from the control group and black circles represent stroke patients. (C) Signed errors for each patient and mean value of the control subjects.

Control participants reported an average signed error of 1.2 ±0.2 cm, while the average error for stroke patients was 0.5±0.4 cm, indicating a higher tendency of undershooting for control participants compared to stroke patients (Fig 3A). However, the results were not significantly different between the two groups, *F*_(1,16)_ = 0.94, *p* = 0.35. Differently from absolute errors, no effect of target on signed errors was detected, *F*_(2,32)_ = 0.48, *p* = 0.62. Mean errors for contralateral, central, and ipsilateral targets were 0.9±0.5 cm, 1.3±0.5 cm and 1.4±0.7 cm for the control group, and 0.6±0.6 cm, 0.6±0.5 cm and 0.3±0.7 cm for the stroke group (Fig 3B). Also no interaction effect was detected, *F*_(2,32)_ = 1.37, *p* = 0.27.

Comparison of each stroke participant with the average control subject revealed a higher error for subjects S2, S4 and S5, as shown in figure (Fig 3C). While subject S2 undershot the target in all the trials, the opposite trend was found for subject S4 which always overshot. The significant differences between control group and stroke participants were found in the contralateral and central targets for patient S2 and in all targets for patient S4, while patient S5 showed a decreased proprioceptive acuity only in the ipsilateral direction (Table 3).

**Table 3:** Pairwise comparisons of signed errors between each subject and the control group in the 3 directions. Mean differences are evaluated as Control-S. Significant differences are marked with an asterisk.

	Contralateral		Central		Ipsilateral	
S	Mean Difference	p-value	Mean Difference	p-value	Mean Difference	p-value
1	1.1	0.06	0.8	0.37	2.2	0.01
2	-3.3	<0.005*	-2.8	<0.005*	-2.3	0.01
3	0.8	0.15	1.9	0.03	-0.6	0.48
4	3.5	<0.005*	3.8	<0.005*	5.9	<0.005*
5	0.8	0.15	1.4	0.11	2.6	<0.005*
6	-0.5	0.36	0.3	0.76	-0.9	0.30
7	-0.3	0.64	1.3	0.13	1.8	0.03
8	0.9	0.14	0.6	0.52	0.8	0.31
9	0.7	0.21	0.1	0.94	-0.3	0.72

### Variability

**Fig 4:**
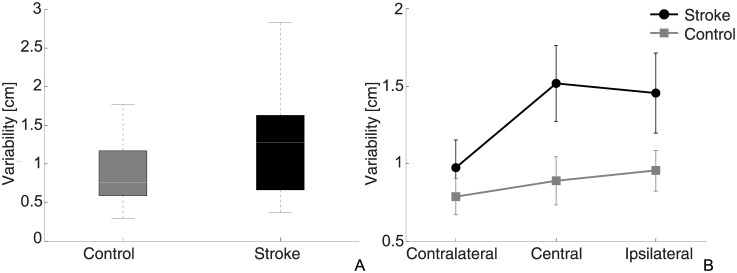
Variability. (A) Box-plot of variability for the two groups. (B) Mean variability in the 3 directions for the two groups, where the gray squares represents data from the control group and black circles represent stroke patients.

Stroke patients showed a more variable estimation of the target position (1.3±0.1 cm) compared to the control group (0.9±0.1 cm), as shown in Fig 4A. However, the statistical analysis failed to detect a significant difference in matching precision between groups, *F*_(1,16)_ = 3.85, p = 0.07. There was a significant effect of target, *F*_(2,32)_ = 4.41, p = 0.02: the lowest variability was found for the contralateral target (control group: 0.8±0.1 cm; stroke group: 1.0±0.2 cm), followed by the central (control group: 0.9±0.2; stroke group: 1.5±0.3) and the ipsilateral target (control group: 1.0±0.1 cm; stroke group: 1.5±0.3 cm). Also for this metric, the interaction between Group and Target was not significant, *F*_(2,32)_ = 1.62, *p* = 0.21. While the central target was identified with similar variability as the ipsilateral (mean difference±SEM: 0.0±0.2 cm, p = 1) and the contralateral (0.3±0.1 cm, *p* = 0.74), the difference between contralateral and ipsilateral matching precision (0.3±0.1 cm) was significant (*p* = 0.01), as revealed by the post-hoc analysis (Fig 4B).

## Discussion

The study investigated the clinical applicability and potential validity of utilizing a passive joint position matching task to evaluate proprioceptive acuity in stroke affected population by means of a low cost planar robotic manipulator. We defined clinical applicability in terms of spectrum of participants (with motor impairment ranging from moderate to severe) that can benefit from the proposed assessment and in terms of time efficiency, i.e. how much time it takes to perform the proposed proprioceptive assessment. Validity was verified in terms of robustness of the proposed approach to differentiate between healthy and stroke participants and its relation to task dependence. In the following paragraphs, we discuss the main findings.

### Clinical applicability

Stroke results in abnormal muscle coactivation of shoulder abductors and elbow flexors of the paretic arm during reaching movements [31] which are marked for individuals with moderate to severe stroke [32–34]. The abnormal coactivation results in a reduced active range of motion, as extensively documented in the work of Beer et al. [33, 35, 36] which would affect the matching performance in an active proprioceptive task. The method proposed in this work allows the assessment of joint position sense independently of the severity of motor impairments as the movement is fully assisted by the robotic device. Patients with the lowest FMA score were, in fact, able to accurately identify the target position even in presence of reduced motor functions (See Figs 2C and 3C). Further, all participants were fully able to understand and complete the proprioceptive assessment, which was inherently simple. Hence the proposed approach potentially is applicable to a large spectrum of population.

Further, due to the modest time required for the proposed assessment (less than 10 min), it can be included in conjunction with conventional motor assessments [37] or robotic assessments, such as the ones proposed by Hussain et al. administrated with the same robotic device [38], or other methods reported in literature [39, 40]. As a consequence, regular assessments can be carried out to detect changes in joint position sense over time, unveiling a more detailed insight into the relationship between training and sensorimotor recovery.

While the proposed procedure avoids non-proprioceptive modalities which could be sources of errors, it cannot rule out the possibility that the measured acuity is affected by a memory component. For this reason, clinicians should prefer different tests when assessing individuals with memory issues, such as a matching task in presence of a persistent visual reference position or a contralateral matching tasks (i.e. opposing limb sets the target configuration throughout the task). This last procedure has, however, the limitation of not being able to ascertain whether the errors are due to noise in the perception of the reference arm position or of the matching arm [41].

### Clinical validity

The observed low intrasubject variability within control participants is consistent with our previous findings on joint position sense of healthy participants in a broader age range [42], making the proposed technique potentially robust for providing a sensitive measure that allows the detection of proprioceptive deficits. Compared to clinical tests (i.e. RASP [43] and NSA [44]), in which proprioceptive tests simply assess the patients’ ability to determine in which direction a joint has been moved or to mirror the change of movement with the unimpaired limb within an error of 10°, the proposed robotic assessment provides a quantitative and automated measure of joint position sense, potentially providing a more repetitive, reliable and sensitive test. Moreover, it could eliminate the problem of low inter-rater reliability which affects the conventional scales.

In this study, we compared the matching error and variability of position estimation of the stroke group with data obtained from age–matched healthy participants. Although control participants had a lower absolute error and variability, we did not observe significant differences between the two groups. This finding suggests that the majority of stroke participants were not different from the control group and therefore had intact proprioception. A closer inspection of data of individual participants revealed that two patients had a significantly different matching performance compared to the mean results of the control group. Namely, subjects S2 and S4 consistently recognized the target position with higher error compared to the mean absolute, and signed errors of the control subjects, while we found a proprioceptive deficit in only one direction for subject S5. This is in accordance with other studies that show that not all patients are affected by proprioceptive deficits: Dukelow et al. found that about 50% of total sub-acute stroke patients examined presented some element of proprioception impairment [21]. In another study, Semrau et al. found that, in a group of 56 patients, there was a trend for the 48% who displayed proprioceptive deficits one week after stroke to relearn proprioceptive skills throughout the course of the first 6 months of recovery [45]. Their finding suggests that a lower percentage of chronic stroke patients are affected by proprioceptive deficits, which is in line with our results.

The two subjects with joint position sense deficits presented a lesion which can generate proprioceptive impairments: S2 had a lacunar stroke with a lesion in the left corona radiata, while S4 had a subcortical stroke involving the right basal ganglia. Corona radiata infarcts have a wide clinical spectrum including incomplete motor and sensory loss [46], and causing sensory disturbances localized to the limbs [47], which also affect proprioception [48]. Basal ganglia are specifically involved in the control of movement amplitude [49], with lesions in this area leading to proprioceptive dysfunction [50, 51].

We also found a trend for stroke patients to identify the target position in a more variable fashion compared to the control group, which is consistent with previous results: Dukelow et al. [21] and Leibowitz et al. [52] found a high variance in repeated trials performance of stroke patients compared to healthy individuals. These studies compared matching performance during a bimanual task, in which the position of the affected hand was matched with the healthy hand. Due to the different methodology, however, a direct comparison of matching errors is not possible: the errors obtained in these studies could arise from both arms, not providing a measure of paretic arm joint position sense, as proprioception of both sides can be affected after unilateral stroke [17, 53] due to disturbances of interhemispheric, transcallosal transfer [54, 55]. However, observed similarities in terms of variability outcomes in the proposed and previous studies serve as a good indicator of the validity of proposed approach.

Another finding supporting the validity of the discussed methodology is that the target position consistently affected the magnitude of the errors of both groups. Proprioception has been found to be not uniform across the workspace, with greater acuity when estimating hand positions closer to the body due to the larger total changes in joint angle associated with hand movements in this area [56]. Matching errors were smaller for the contralateral target: this concurs with results published in a review by Goble [27] which reported matching errors measured for right-handed healthy subjects when matching targets in the right or left workspace. The study found a better performance when matching positions in the left of the body midline (contralateral), especially in the far-left workspace. Also the study of Haggard et al. revealed a contralateral advantage in matching task for right-handed participants, with smaller matching errors for positions in the extreme-left workspace [57].

A possible explanation for this is that movements towards contralateral and central targets require the coordinated rotation of shoulder and elbow joints, while for the ipsilateral target, single joint movements of either shoulder or elbow joints are required [58]. The stretch of muscle spindles is directly related to joint angle change [56] and while signals from two joints are combined when perceiving movements towards central and contralateral targets, movements toward the ipsilateral side are mainly signaled from the rotation of one joint. Moreover, movements toward the contralateral target caused the shoulder to approach the limit of movement, causing tension in muscles, capsuloligamentous structures, and skin. Passive stretch of the muscles near the end of the ROM may result in the enhancement of the number of activated muscle spindles resulting in the improvement of the position sense acuity. Indeed, it has been demonstrated that joint position sense is more accurate near the end of the ROM of the shoulder [59] and the wrist [60] where there is more tension and restraints of motion.

### Limitations

A limitation of the current study is the small sample of patients examined. For this reason, no relationship between motor and proprioceptive function can be drawn from our results; it is worth anyway to highlight that the two stroke patients (S2 and S4) that performed more poorly than control subjects have some of the highest FMA scores. The relationship between FMA scores and matching performance with the robotic test will be object of future studies.

Moreover, patients were recruited for a randomized control trial evaluating the feasibility of using a robotic device for motor training. The inclusion criteria limited the spectrum of motor and sensory impairments which are present in the stroke population. Due to the lack of clinical scores on joint position sense in this study, we could not establish the correlation between the robotic assessment and the conventional tests. Furthermore, sensitivity and specificity of the proposed test have yet to be determined with a larger number of patients.

A disadvantage of the current paradigm is the addition of delays imposed by having the experimenter pressing the button after the subject verbally reports the reaching of the target position. These delays might introduce noise and bias in the results, which, however, had a limited overall effect on the evaluated position variability (1.3 cm and 0.9 cm for patients and healthy participants respectively) due to the low velocity of the robot. In fact, the reaction time to auditory cues has been documented to be 140-160 ms [61], which would result in an error of about 0.3 cm in the position estimation. An alternative approach to avoid this error is to let the patient press the button. In this experimental condition, the task would become bimanual, requiring transcallosal transmission from the primary somatosensory region of the cerebral cortex in the contralateral hemisphere to control the action of the unimpaired limb. The interhemispheric transfer would add an additional cognitive factor which may influence the position matching performance.

A future study will further validate the methodology with a larger cohort of stroke affected population presenting a larger spectrum of impairments. This study will help in establishing normative values of joint position sense acuity that will allow the identification of proprioceptive deficits. The proposed assessment will help therapists in selecting the most appropriate therapy tailored to the patients’ need.

## Conclusion

There is a clear distinction between research-oriented measures of proprioception and those used in clinical settings. Technologies employed to obtain the former measures allow for more precise, accurate, and reliable assessments, particularly in terms of limiting the influence of factors other than proprioception on the outcome; however, the clinical applicability associated with these measures is usually limited due to the amount of equipment required, technical expertise and time required for their administration. As an example, although the psychometric methods have high sensitivity, they require an extensive amount of time and precise manipulation of limb joints for their implementation, hence limiting their applicability in clinical environments. In contrast, current clinically friendly tests that are conventionally used have very limited sensitivity due to ordinal nature. In this study, we propose a sensitive measure (based on integrated sensors for high precision assessment) of joint position sense which can be translated into clinical practice, due to the short time required for assessment and further, it can be administrated with the same robotic technology designed for motor rehabilitation tasks. Future studies will investigate the applicability of such measure for assessing the effect of rehabilitation programs on proprioception and to draw correlation between clinical sensory testing and the robotic measures.

## Supporting information

S1 Dataset Signed errors of healthy participants. (XLSX)

S2 Dataset Signed errors of stroke patients. (XLSX)
